# The Antecedents and Consequences of Health Literacy in an Ecological Perspective: Results from an Experimental Analysis

**DOI:** 10.3390/ijerph15040798

**Published:** 2018-04-19

**Authors:** Chiara Lorini, Francesca Ierardi, Letizia Bachini, Martina Donzellini, Fabrizio Gemmi, Guglielmo Bonaccorsi

**Affiliations:** 1 Department of Health Science, University of Florence, Viale GB Morgagni 48, 50134 Firenze, Italy; guglielmo.bonaccorsi@unifi.it; 2 Quality and Equity Unit, Regional Health Agency of Tuscany, 50141 Florence, Italy; francesca.ierardi@ars.toscana.it (F.I.); letizia.bachini@ars.toscana.it (L.B.); fabrizio.gemmi@ars.toscana.it (F.G.); 3 School of Specialization in Hygiene and Preventive Medicine, University of Florence, 50134 Florence, Italy; martinadonzellini@gmail.com

**Keywords:** health literacy, ecological study, antecedents, consequences, determinants of health

## Abstract

This study analyses the relationship between the antecedents and consequences of health literacy (HL) at the ecological level among the nations involved in the European Health Literacy Survey (HLS-EU). The antecedents and consequences were investigated by means of proxy indicators. The HL was measured using the 47-item HLS-EU questionnaire (HLS-EUQ47) and the Newest Vital Sign (NVS). The two measures stood in significant correlation to the outcomes of the sub-discipline of the Euro Health Consumer Index (r = 0.790 for HLS-EUQ47; r = 0.789 for NVS). The HLS-EUQ47 also stood in correlation to the percentage of population with post-secondary education (r = 0.810), the reading performance for 15-year-old students (r = 0.905), the presence of a national screening program for breast (r = 0.732) or cervical cancer (r = 0.873). The NVS stood in correlation with the unemployment rate (r = −0.778), the Gross Domestic Product (r = 0.719), the Gini coefficient (r = −0.743), the rank of the Euro Patient Empowerment Index (r = −0.826), the expenditure on social protection (r = 0.814), the Consumer Empowerment Index (r = 0.898), the percentage of adults using the internet for seeking health information (r = 0.759), the prevalence of overweight individuals (r = −0.843), the health expenditure (r = 0.766), as well as the percentage of individuals using the internet for interacting with public authorities (r = 0.755). This study provides some preliminary considerations regarding alternative means by which to study HL and proposes new methods for experimentation. The methods and the results could offer a means by which the relationship between society and overall healthcare protection could be strengthened.

## 1. Introduction

Health literacy (HL) is a multifaceted concept that concerns the capacities of individuals to meet the complex demands of health in a modern society [1].

With regard to research and practice in terms of HL, two approaches have predominated during recent decades: the individual (clinical) level and the public health level. The first approach is the oldest: it focuses on an individual’s capacity to obtain, process, and understand basic health information, including health services, needed to make appropriate health-related decisions. This approach highlights existing gaps within strategies of treatment, prevention, and health promotion as well as overall health behavior, including specific individual health-related outcomes [2]. The second approach incorporates knowledge as to the social determinants of health and relates to the definition of public health literacy, “the degree to which individuals and groups can obtain, process, understand, evaluate, and act upon information needed to make public health decisions that benefit the community” [3,4].

In terms of the public health perspective, Sørensen et al. [5] proposed a comprehensive model with an integrated definition: “Health literacy is linked to literacy and entails people’s knowledge, motivation and competences to access, understand, appraise, and apply health information in order to make judgments and take decisions in everyday life concerning healthcare, disease prevention and health promotion to maintain or improve quality of life during the life course”. The conceptual framework proposed by Sørensen identified four dimensions of HL (access, understand, process, and apply) which could be applied to three domains (health care, disease prevention, health promotion). The framework also took into consideration the proximal and distal factors (antecedents) which impact HL as well as its related outcomes (consequences). Within Sørensen’s framework, antecedents specifically refer to societal and environmental factors (i.e., demographics, culture, language, political forces, societal systems) as distal factors whereas situational determinants (i.e., social support, family and peer influence, media use, physical environment) and personal determinants (i.e., age, gender, race, socioeconomic status, education, occupation, employment, income, literacy) are considered proximal factors. Consequences at both the individual and population level refer to health service use and health costs; health behaviour and health outcomes; participation and empowerment in health issues; equity and sustainability of public health issues. Such a framework suggests two levels of analysis and intervention, the subject level and the ecological level. Sørensen’s model has been used as a basis for developing the multidimensional questionnaire used to measure and compare HL in the general population (the HLS-EU-Q) of eight European countries in the European Health Literacy Survey (HLS-EU) [6,7]. To date, it has been the first attempt to measure HL in different countries at the same time using the same measures.

Many studies have shown the correlation between antecedents and HL, as well as between consequences and HL [8,9,10,11]. The majority of such studies focused on only a few similar or correlating factors; this has led to a fragmentation of the results without an overall quantitative assessment of the relationship among all relevant factors.

To the best of our knowledge, no studies have been published regarding the ecological relationships between HL and its antecedents and consequences in terms of macro-level factors. Such information could guide policy makers in providing appropriate responses to the needs of citizens. As such, this study identifies a set of indicators, available using free data from international databases or from published documents, to test according to an ecological model. Accordingly, this paper provides a novel approach to the study of health literacy. This paper aims to advance our understanding of the relationship among nationally determined contextual characteristics within the countries included in the HLS-EU in terms of their role as HL antecedents or consequences.

## 2. Materials and Methods

The study objective was addressed using an ecological model in which the antecedents and consequences of HL were measured at country level. The design of the study was suitable to investigate macro-level properties, namely political, economic, demographic, and health contexts, through proxy indicators.

### 2.1. HL (Health Literac) Measurements

Data on HL were obtained through consultation of the published results of the first HLS-EU, conducted in 2011 in eight countries (Austria, Bulgaria, Germany, Greece, Ireland, the Netherlands, Poland, and Spain) [7,12,13]. In this survey, HL was measured by means of two tools: the HLS-EUQ47 and the Newest Vital Sign (NVS). The first consisted of 47 items comprising the core of the HL model, a twelve-cell matrix positing the key processes of accessing, understanding, appraising, and applying health-related information within three domains (healthcare, disease prevention, health promotion) [5,6]. According to Nutbeam’s definition [14], it assessed functional, interactive, and critical HL (Table 1).

For each item, respondents rated the perceived difficulty of a given task, resulting in a subjective assessment of HL. The answers were placed on a four-category Likert scale (from “very easy” to “very difficult”) then converted into a score. Using the scores of the 47 items, the authors constructed a comprehensive general index of HL (total score ranging from 0 to 50) which was used to define the ranges for different levels of HL (“inadequate”, “problematic”, “sufficient”, “excellent” general-HL).

The NVS is a rapid assessment instrument for measuring functional HL, including numeracy. It assesses the respondents’ ability to read and apply information from a nutritional label for ice cream and constitutes an objective assessment of HL [15]. The UK version of the NVS [16], which was used in the HLS-EU, consisted of seven questions related to the nutritional label. According to the number of correct answers (from 0 to 6), a raw score was computed indicating the likelihood of a level of HL (“high likelihood of limited literacy”, “possibility of limited literacy”, “high likeliness of adequate literacy”).

Literature data [7,13] report both the descriptive statistics of the total score and the levels of HLS-EU-Q47 and of NVS by country; however, for this study, only mean values of HLS-EU-Q47 and of NVS were considered.

### 2.2. Antecedents and Consequences of HL

The final set of antecedents and outcome indicators was identified following a three-stage approach. First, Sørensen review [5] was used to define antecedents and consequences by area. Then, a literature review was conducted to select a list of indicators related to antecedents and consequences according to the different areas. Finally, the availability of the listed indicators for the eight countries involved in the HLS-EU was verified via international databases and documents.

The literature review was conducted through a Pubmed search of ecological studies conducted at the national level, including studies which analysed any aspect of health. Moreover, web-available documents issued by international organizations focused on international comparisons describing health or health-related indicators at the national level were searched and reviewed. Selected documents were analyzed to identify all indicators used and a list of these indicators was compiled.

Subsequently, the availability of each of the listed indicators was checked. Aggregate country-level antecedent and consequence indicator data were extracted from several databases and/or documents which was available from the websites of Eurostat, European Health for All (HFA-DB), the Organisation for Economic Co-operation and Development (OECD), the Health Consumer Powerhouse (HCP), the World Health Organization, and the European Commission. To obtain the most reliable information for comparison with the HL average, data was considered adequate and included in analysis if it referred to the three-year period preceding the HLS-EU (2009–2011). When data were available which referred to more than one year within the three-year period, those relating to the year closest to the HLS-EU were included in the analysis. Finally, indicators were included in the analysis only when available for each of the eight countries included in the HLS-EU. If an area was over-represented in the final database (i.e., with more than five indicators), the less frequently used indicators for international comparison were omitted.

### 2.3. Statistical Analysis

Using each country as a unit of analysis, a correlation analysis was performed by means of Spearman rank correlation coefficients, which included the final set of indicators and the HL measures (HLS-EU-Q47 and NVS mean scores).

The analysis was conducted using STATA, release 12.1 (StataCorp LLC, College Station, TX, USA). Statistical significance was set at *p* = 0.05.

## 3. Results

### 3.1. The Selection of the List of Indicators

According to Sørensen’s review, he antecedents and consequences by areas are listed in Table 2. Since health outcomes could be considered both antecedents and consequences of HL, this area was included in both sections.

Literature review led to the selection of eight ecological studies [17,18,19,20,21,22,23,24] and 14 documents [25,26,27,28,29,30,31,32,33,34,35,36,37,38], generating a list of approximately 250 indicators (Table S1). As shown in Table 1, some areas were not represented by any indicator since they were not investigated in the selected studies. Most of the indicator data were not available in the consulted databases or documents for every county included in the HLS-EU in the period 2009–2011.

The final list of indicators (*N* = 37) included in the analysis as well as the data sources is reported in Table 3. Table S2 contains the final dataset.

### 3.2. The Correlation Analysis

The HLS-EUQ47 and the NVS mean scores are not significantly correlated (r = 0.419; *p* = 0.301).

Table 4 reports the results of the correlation analysis. The HLS-EUQ47 and the NVS scores present different results. With regard to antecedents, the HLS-EUQ47 mean score stood in significant correlation to the percentage of population with post-secondary education (r = 0.810), reading performance for 15-year-old students (r = 0.905), the presence of a national breast cancer screening program (r = 0.732), and the presence of a national cervical cancer screening program (r = 0.873). Regarding consequences, the mean score stood in significant correlation to only the “outcomes” sub-discipline of the Euro Health Consumer Index (r = 0.790).

In terms of the NVS mean score, significant correlations existed among the unemployment rate (r = −0.778), the Gross Domestic Product (GDP, r = 0.719), the Gini coefficient (r = −0.743), the rank of the Euro Patient Empowerment Index (EPEI) total score (r = −0.826), and the expenditure on social protection (r = 0.814) as antecedents. Moreover, the NVS mean score was also significantly associated with the Consumer Empowerment Index (r = 0.898), the percentage of adults using the internet for seeking health information (r = 0.759), the prevalence of overweight individuals (r = −0.843), the “outcomes” sub-discipline of the Euro Health Consumer Index (r = 0.789), the total health expenditure (r = 0.766), and the percentage of individuals using the internet for interacting with public authorities (r = 0.755) as consequences.

## 4. Discussion

This study investigates the ecological relationships between the antecedents and consequences of HL as related to macro-level factors. To the best of our knowledge, no previous ecological studies on HL have previously been published. Accordingly, comparisons with other studies are not possible. On the other hand, many studies have explored the relationships the antecedents and consequences of HL at the individual level.

According to other researchers [39,40,41], HL is not only an individual variable but also a social practice. It is a distributed resource (distributed HL) within an individual’s social network, where health literate subjects share their HL skills to support other individuals as to how to manage their health, communicate with health professionals, and make overall decisions about their health. Batterham et al. [41] stressed the importance of a distributed HL both for individual empowerment (freedom of choices and participation in decision making) and adherence to professional medical advice. Accordingly, the study of HL as an ecological variable allows us to better understand the role of this determinant of health.

Ecological design is appropriate if researchers are interested in the effect of macro-level aspects. As such, this study could be a valid contribution in terms of the concept of HL, particularly on the level of national public health. However, this type of study is potentially susceptible to ecological fallacy which can encompass several potential biases: ecological confounding, model specification bias, and ecological bias. Nevertheless, many researchers are confident that this type of study can contribute to creating reliable causal relationships [42].

Sørensen’s integrated conceptual model of HL describes its predominant antecedents and consequences, which resulted from reviewing existing HL concepts [5]. HLS-EU has contributed to a validation of the conceptual model, collecting individual data in eight countries using a comprehensive questionnaire that featured two measures of HL (HLS-EU-Q47 and NVS) and 39 items referring to antecedents and consequences outlined in the conceptual model [7]. Our research further contributes a validation of the conceptual model at the national level. In the HLS-EU-Q, the identification of the 39 items to be included in the questionnaire is the result of a literature review; in our study, the identification of the indicators to be included in the correlation analysis is the result of a literature review as well. As this is the first study which analysed HL at an ecological level, the list of indicators was selected those used in various ecological studies and were attributable to antecedents or consequences as outlined in the Sørensen conceptual model. Accordingly, this study could be described as an experiment to validate the Sørensen conceptual model of HL at an ecological level and an analysis of indicators that are applicable at the national scale. Unfortunately, data availability for the eight countries involved in the HLS-EU and which referred to the three years preceding the HLS-EU limited the possible number of indicators to be entered into the analysis.

No previous ecological studies have been published with either this level of focus on indicators or which have incorporated this many data sources. Indeed, the aim of this study was to identify novel sources of “ecological” data, combining information from international databases (Eurostat, Health for All, and OECD databases) and ad hoc surveys (HLS-EU, Eurobarometer, Eurostat, European Commission, and HCP surveys, Joossens’ study).

Significant amounts of secondary data, already collected or produced by other researchers, are available for free online; this is an excellent opportunity for research, especially for emergent ecological studies. Information provided by databases associated with international organizations can usually be easily obtained via their websites; data from ad hoc surveys are usually described in the results of the studies or can be requested directly from the researchers. Nonetheless, the use of secondary data presents several limitations that could have influenced the quality of this study. Data are neither specific to the aims of this study (fitness for use), nor controlled for quality by the Authors of this study (the Authors are not responsible for primary data). The use of numerous data sources as well as the inclusion of eight nations could have reinforced these critical quality issues.

International databases are frequently compiled from various sources; they are validated and processed in a uniform way to improve the international comparability of statistics. Quality of data is a central issue in the production of health indicators for international organizations; they have quality management policies and they constantly review both their data sources and methodologies. Statistics are checked for consistency, coherence, and comparability [43,44,45]; however, their quality is primarily influenced by the quality of each respective nation’s statistics. Additionally, for some indicators (e.g., migration statistics), a lack of international comparability is a well-known issue [46]. Moreover, the comparability and the accuracy of data reported in the international databases is limited in some cases, owing to a variety of factors including differences in definitions and/or time periods, incomplete registration, or other variations in national data recording and/or processing. Ad hoc surveys can help to overcome the limitations of internal consistency and comparability as a shared study protocol often can be generally applied across all research units. However, even these may present limitations on results due to differing sampling procedures which influence comparability across countries. Moreover, ad hoc studies are limited in time (i.e., data are not routinely collected). On the other hand, such studies usually are innovative and experimentally tentative. Occasionally, pilot exercises to describe macro-level aspects use novel indicators to compare the same phenomena in different countries. Our research may be comparable to such studies; a tentatively novel methodology to integrate significantly different sources of both routine and innovative indicators.

The results of our study are not exhaustive nor conclusive resulting from limitations in the selection of indicators (literature review not related to ecological studies on HL, lack of data availability, some areas outlined in the Sørensen model not represented in our study) as well as in the quality and comparability of some indicators. Moreover, the study design and the low number of countries involved (eight) limited the statistical analysis and the strength of the results. For example, correlation analysis is sensitive to outliers; although Spearman’s correlation is less sensitive to outliers, the low number of observations may have influenced the results [47].

Despite these limitations, some tentative conclusions may be drawn from the results. This study provides some preliminary regarding the antecedents and consequences of HL which require additional analysis.

The HLS-EUQ47 and the NVS scores showed no significant correlation and presented different results in the correlation analysis. The data analysis of the HLS-EU, conducted at the individual level, showed a significantly positive but low correlation between the HLS-EU-Q47 and the NVS scores, with a coefficient equal to 0.25 [13]. The relationship between objective and subjective HL measures has received limited attention. Few studies using multiple instruments have been conducted to date [48]. At a conceptual level, these tools measure different constructs: the Sørensen definition of HL for the HLS-EUQ47 [5], that of the U.S. Department of Health and Human Services [49] for the NVS. The NVS provides a measure of individual HL, which is the consequence of both the individual skills and the complexity of the context within which people act [50]. In contrast, the HLS-EUQ47 is a measure of public HL [51]. Since they measure different aspects of HL in different ways, it is not surprising that they led to different results in this study; however, the two measurements provided a more complete picture of HL.

Without separating the results which emerged from the two different measurement tools, our data show that HL is related to the following antecedents on an ecological (national) level: the percentage of the population with post-secondary education (r = 0.810 with HLS-EUQ47 score), the reading achievement (r = 0.905 with HLS-EUQ47 score), the unemployment rate (r = −0.778 with NVS score), the GDP (r = 0.719 with NVS score), the Gini coefficient (r = − 0.743 with NVS score), the presence of a national breast cancer screening programme (r = 0.732 with HLS-EUQ47 score) or of a national cervical cancer screening programme (r = 0.873 with HLS-EUQ47 score), the national rank of the EPEI total score (r = −0.826 with NVS score), and the expenditure on social protection (r = 0.814 with NVS score). Surprisingly, demographic data (indicators related to gender, age, or ethnicity distribution) showed no correlation with HL. Moreover, HL stood in correlation to the following consequences: the Consumer Empowerment Index (r = 0.898 with NVS score); the percentage of adults using the Internet for seeking health information (r = 0.759 with NVS score); the prevalence of overweight (r = −0.843 with NVS score); the outcome sub-discipline of the Euro Health Consumer Index (r = 0.790 with HLS-EUQ47 score, r = 0.789 for NVS score); the total health expenditure, as percentage of GDP (r = 0.766 with NVS score); and the percentage of individuals using the Internet for interacting with public authorities (r = 0.755 with NVS score).

Accordingly, national policies devoted to promote and provide the prerequisites of health (specifically education, income, social justice, and equity), to increase health coverage (i.e., the introduction of national screening programmes), and to make healthcare systems more empowering for the patients should result in a widespread increase of HL among a nation’s population. On the other hand, those policies (particularly those dedicated to increasing functional HL) should contribute to the following results: an increase in consumer empowerment, the decrease of the prevalence of overweight individuals, the increase of the health status of the population, and the increase of total health expenditure. It is important to highlight that the increase of consumer empowerment as well as the decrease of the prevalence of overweight individuals and general obesity are among the main objectives of the European Commission [52,53]. Moreover, the results suggest that, where the HL of the population is high, the Internet could be used effectively by policy makers and experts for the provision of information and services related to health and health services. It may also provide a means by which to interact with the population. In contrast, in countries where the HL of the population is low, such tactics may contribute to the digital divide [54], increasing overall inequality.

## 5. Conclusions

This study provides some preliminary considerations regarding different approaches to study HL including the potential of analyzing the ecological level of HL as well as other novel methods of analysis. It also provides a list of indicators by which one may validate the Sørensen conceptual model using secondary data. Both the methods and the results need to be analysed further; however, both will offer, when weaknesses and limits are reduced, a key method to strengthen the relationship between society and healthcare protection.

## Figures and Tables

**Table 1:** Health literacy (HL) definition, according to Nutbeam [14].

Functional HL	Basic Reading, Writing, and Literacy Skills
Interactive HL	Communicative and social skills that can be used to derive meaning from different forms of communication, and to apply new information to changing circumstances
Critical HL	Cognitive and social skills required to critically analyse information, and to use this information to exert greater control over life events and situations through individual and collective action to address the social, economic and environmental determinants of health

**Table:** A—Antecedents.

Levels	Areas	Sub-Areas
Personal Determinants (Proximal Factors)	Demographic	Age
		Gender
		Race/ethnicity
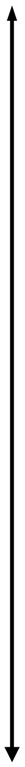	Competences	Literacy
		Education level
		Operational competences
		Interactive competences
		Autonomous competences
		Informational competences
		Contextual competences
		Cultural competences
		Media use
		Peer and parent influences
		Reading and arithmetical skills
	Socioeconomic	Occupation
		Employment status
		Income
		Income discrepancy
	Health	Disease severity
		Health status
		Health-related experience
		Personal competences such as vision, hearing, verbal ability, memory and reasoning
		Cognitive abilities
		Physical abilities
	Healthcare	Health coverage
		Health system
		Communication and assessment skills of people with whom individuals interact for health
		Complexity and difficulty of the printed and spoken messages in the healthcare environment
Situational Determinants (Distal Factors)	Policy	Health promotion actions (education, social mobilization, advocacy)
		Ability of the media, the marketplace, and governmental agencies to provide health information in an appropriate manner
		Social support
		Education system
		Social, environmental and political forces

**Table:** B—Consequences.

Levels	Areas	Sub-Areas
Individual	Health outcomes	Health status (also self-reported)
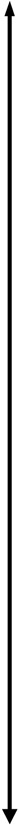		Health outcome
	Health behavior	Health behaviors
		Prevention behaviors
		Health-promoting behaviors
		Screening behaviors
		Compliance
		Medical or medication treatment errors
	Empowerment	Attitudes
		Self-efficacy
		Capacity to act independently on knowledge
		Motivation and self-confidence
		Individual resilience
		Ability to apply information to novel situations
		Self-management skills/ability to care
		Health knowledge (risk, diseases and treatments)
		Improved capacity to influence social norms and interact with social groups
		Improved capacity to act on social and economic determinants of health
	Participation	Ability to participate in public and private dialogues about health, medicine, scientific knowledge and cultural beliefs
		Patients-provider interactions
		Screening behaviors
	Health services use	Hospitalization
		Emergency care
		Use of healthcare services
		Healthcare access
	Sustainability	Social injustice
		Healthcare costs
	Equity	Healthcare access
		Improved capacity to influence social norms and interact with social groups
		Improved capacity to act on social and economic determinants of health
Community/Social		Social injustice

**Table 3:** Indicators included in the correlation analysis and data sources.

Sub-Area	Indicator	Data Source	Year
**Antecedents**			
Gender	Women/100 men	Eurostat	2011
Age	Population aged 65+ (%)	Health for All	2011
Race/ethnicity	Foreign-born population (%)		2011
Education level	Population with post-secondary education aged 25+ (%)	Health for All	2010
Education level	Lifelong learning-% persons aged 25 to 64 who stated that they received education or training in the four weeks preceding the survey	OECD	2011
Media use	Population that use the Internet at least ones a week (% of individuals)	Eurostat	2010
Reading and arithmetical skills	Reading achievement (average reading performance for 15-year-old students)	OECD	2009
Employment status	Unemployment rate (%)	Health for All	2011
Income	Gross Domestic Product (GDP), U.S.$ per capita	Health for All	2011
Income discrepancy	Gini coefficient	Health for All	2011
Health status	Prevalence of chronic depression (%)	OECD	2011
cognitive abilities	Prevalence of dementia (%)	OECD	2009
Health coverage	National breast cancer screening program (0 if not, 1 if yes)	European Commission	2011
Health coverage	National cervical cancer screening program (0 if not, 1 if yes)	European Commission	2011
Health systems	Hospitals per 100,000 abitants	Health for All	2011
Health systems	Euro Patient Empowerment Index-total score-rank	HCP	2009
Health promotion actions	Tobacco Control Scale Ranking	Joossens, 2013	2010
Social support	Expenditure on social protection (% of GDP)	Eurostat	2011
Social, environmental and political forces	Households with internet access (%)	Eurostat	2011
**Consequences**			
Capacity to act independently on knowledge, Motivation and self-confidence, individual resilience, Ability to apply information to novel situations, Ability to participate in public and private dialogues about health, medicine, scientific knowledge and cultural beliefs, self-efficacy, attitudes	Consumer Empowerment Index score	Eurobarometer	2011
Health knowledge (risk, diseases and treatments)	Individuals (16–74) using the internet for seeking health information (%)	Eurostat	2011
Health behavior	Prevalence of overweight (%)	Health for All	2010
Health behavior	Pure alcohol consumption (litres per capita)	Health for All	2011
Health behavior	Adult population smoking daily (%)	OECD	2010
Health outcomes	Life expectancy at birth	Eurostat	2011
Health outcomes	Suicide and self-inflicted injury death rate per 100,000 abitants	Health for All	2011
Health status	Self-perceived health-% Bad	Eurostat	2011
Health outcomes	People having a long-standing illness or health problem by educational attainment level	Eurostat	2011
Health outcomes	Euro Health Consumer Index-Outcomes sub-discipline	HCP	2009
Hospitalization	Hospital discharges per 1000 inhabitants	OECD	2010
Healthcare costs	Total health expenditure (% of GDP)	Health for All	2011
Healthcare costs	Private expenditure on health as % of total expenditure on health	Health for All	2011
Healthcare access	Self-reported unmet need for medical examination or treatment	Eurostat	2011
Screening behaviors	Mammography screening, women aged 50–69 screened (%)	OECD	2010
Improved capacity to influence social norms and interact with social groups	Individuals using the internet for interacting with public authorities (last 12 months) (%)	Eurostat	2011
Social injustice	Crime, violence or vandalism in the area	Eurostat	2011
Social injustice	UNDP Human Development Index (HDI)	Health for All	2011

HCP: Health Consumer Powerhouse; OECD: Organisation for Economic Co-operation and Development; UNDP: United Nations Development Programme.

**Table 4:** Spearman’s correlation between HL (mean value of HLS-EU-Q47 and NVS scores), its antecedents and its consequences in the eight European countries of the HL Survey.

Indicators		HLS-EUQ 47 Score		NVS Score	
		Rho	*p*	Rho	*p*
Antecedents	Women/100 men	−0.611	0.108	−0.614	0.105
	Population aged 65+ (%)	−0.405	0.320	0.168	0.691
	Foreign-born population (%)	0.262	0.531	0.275	0.509
	Population with post-secondary education aged 25+ (%)	0.810	0.015	0.635	0.091
	Lifelong learning-% persons aged 25 to 64 who stated that they received education or training in the four weeks preceding the survey	0.381	0.352	0.551	0.157
	Population that use the Internet at least ones a week (% of individuals)	0.667	0.071	0.647	0.083
	Reading achievement (average reading performance for 15-year-old students)	0.905	0.002	0.299	0.471
	Unemployment rate (%)	−0.071	0.867	−0.778	0.023
	GDP, U.S.$ per capita	0.667	0.071	0.719	0.045
	Gini coefficient	−0.524	0.183	−0.743	0.035
	Prevalence of chronic depression (%)	0.275	0.509	0.590	0.123
	Prevalence of dementia (%)	0.095	0.823	0.168	0.691
	National breast cancer screening program (0 if not, 1 if yes)	0.732	0.039	−0.113	0.789
	National cervical cancer screening program (0 if not, 1 if yes)	0.873	0.005	0.274	0.511
	Hospitals per 100,000	−0.595	0.120	0.000	1.000
	EPEI total score-rank	−0.595	0.120	−0.826	0.011
	Tobacco Control Scale Ranking	−0.518	0.188	0.248	0.553
	Expenditure on social protection (% of GDP)	0.381	0.352	0.814	0.014
	Households with internet access (%)	0.286	0.493	−0.036	0.933
Consequences	Consumer Empowerment Index	0.548	0.160	0.898	0.002
	Individuals (16–74) using the internet for seeking health information (%)	0.407	0.317	0.759	0.029
	Prevalence of overweight (%)	−0.024	0.955	−0.843	0.009
	Pure alcohol consumption, litres per capita	0.072	0.866	−0.548	0.159
	Adult population smoking daily	−0.476	0.233	−0.575	0.136
	Life expectancy at birth	0.238	0.570	0.228	0.588
	Suicide and self-inflicted injury (SDR) per 100,000	0.048	0.911	0.060	0.888
	Self-perceived health-% Bad	−0.595	0.120	−0.275	0.509
	People having a long-standing illness or health problem by educational attainment level	0.024	0.955	−0.530	0.177
	Euro Health Consumer Index-Outcomes sub-discipline	0.790	0.020	0.789	0.020
	Hospital discharges per 1000 inhabitants	−0.524	0.183	0.228	0.588
	Total health expenditure (% of GDP)	0.095	0.823	0.766	0.027
	Private expenditure on health as % of total expenditure on health	−0.455	0.257	−0.602	0.114
	Self-reported unmet need for medical examination or treatment	0.371	0.365	0.566	0.143
	Mammography screening, women aged 50–69 screened (%)	0.381	0.352	0.419	0.301
	Individuals using the internet for interacting with public authorities (last 12 months) (%)	0.548	0.160	0.755	0.031
	Crime, violence or vandalism in the area	−0.286	0.493	0.323	0.435
	UNDP Human Development Index (HDI)	0.071	0.867	0.443	0.272

Note: UNDP: United Nations Development Programme.

## Supplementary Materials

The following are available online at http://www.mdpi.com/1660-4601/15/4/798/s1, Table S1: Conceptual model of HL and indicators reported in ecological studies/reports/documents by area. Table S2: Database.
