# Supplementary material for: The Antecedents and Consequences of Health Literacy in an Ecological Perspective: Results from an Experimental Analysis
Source: Int J Environ Res Public Health. 2018 Apr 19;15(4):798. doi: 10.3390/ijerph15040798 (PMC5923840; doi:10.3390/ijerph15040798)
Supplement: Supplementary file 1 [file ijerph-15-00798-s001.zip › ijerph-286786-suppl/ijerph-286786-Table S1.docx]

**Table S1.** Conceptual model of HL and indicators reported in ecological studies/reports/documents by area.

A—Antecedents.

| **Level** | **Area** | **Sub-area** | **Indicators** |
| --- | --- | --- | --- |
| Personal determinants (proximal factors) | Demographic | Age | Median age [20] |
|  |  |  | Population by age [23,24] |
|  |  |  | Birth rate [23] |
|  |  |  | Fertility rate [23] |
|  |  |  | Population projections [23] |
|  |  | Gender | Women (%) [18] |
|  |  |  | Gender distribution [24] |
|  |  | Race/ethnicity/ethnic composition of the community | Foreign-born population [28] |
|  | Competences | Literacy | Adult literacy rate, % of population [19] |
|  |  | Education level | School enrollment (%), tertiary [19,24] |
|  |  |  | Mean years of schooling [20] |
|  |  |  | Prevalence of persons age 18 years currently in school [21] |
|  |  |  | Population by 4 ISCE (International Standard Classification of Education) education classes [23] |
|  |  |  | Age-standardised percentage of people aged 25–64 with less than tertiary (university-level) education, by gender [25] |
|  |  |  | Life-long learning [38] |
|  |  | Operational competences | - |
|  |  | Interactive competences | - |
|  |  | Autonomous competences | - |
|  |  | Informational competences | - |
|  |  | Contextual competences | - |
|  |  | Cultural competencies | - |
|  |  | Media use | %population that use the Internet at least ones a week [27] |
|  |  | Peer and parent influences | - |
|  |  | Reading and arithmetical skills | Reading achievement (average reading performance for 15-year-old students) [26] |
|  | Socioeconomic | Occupation | Population by occupational class, by ISCO (International Standard Classification of Occupations) groups [23] |
|  |  | Employment status | Unemployment, % of male labor force [19] |
|  |  |  | Labor force participation rate [20] |
|  |  |  | Unemployment rate [21] |
|  |  |  | Total employment rate 15-64 [23] |
|  |  |  | Total employment [23] |
|  |  |  | Unemployment rates by duration [25] |
|  |  | Income | Gross Domestic Product (GDP) based on purchasing power parity [18,25] |
|  |  |  | Gross Domestic Product [19,27] |
|  |  |  | Gross National Income [20] |
|  |  |  | Multidimensional Poverty Index [20] |
|  |  |  | Age-standardised percentage of people aged 25 and over by severity of material deprivation [25] |
|  |  |  | Difficulties experienced in paying bills [25] |
|  |  |  | Net disposable income, purchasing power standard based on final consumption per inhabitant [25] |
|  |  | Income discrepancy | Gini coefficient [19,20,25] |
|  |  |  | % population with income below 60% national median [25] |
|  |  |  | Slope index of inequality [25] |
|  |  |  | Relative index of inequality [25] |
|  | Health | Disease severity | - |
|  |  | Health status | UNAIDS (United Nations Programme on HIV/AIDS) global HIV indicators [17] |
|  |  |  | Homicide rate [20] |
|  |  |  | Healthy Life Years expectancy at birth stratified by sex [21,25] |
|  |  |  | Incidence rate of type 1 diabetes amongst children ≤ 14 years of age [22] |
|  |  |  | Death rates from infectious and parasitic diseases, age-standardized [22] |
|  |  |  | Death rates from respiratory infections, age-standardized [22] |
|  |  |  | Bacterial antibiotic susceptibility [22] |
|  |  |  | Life expectancy at various ages [23] |
|  |  |  | Life expectancy of men and women [25] |
|  |  |  | Infant mortality [23,25] |
|  |  |  | Perinatal mortality [23] |
|  |  |  | Standardised death rate, Eurostat 65 causes, age 0-65 [23] |
|  |  |  | Standardised death rate, Eurostat 65 causes, age 65+ [23] |
|  |  |  | Smoking-related deaths [23] |
|  |  |  | Alcohol-related deaths [23] |
|  |  |  | Drug-related deaths [23] |
|  |  |  | Incidence/prevalence of HIV/AIDS [23] |
|  |  |  | Incidence/prevalence of lung cancer [23] |
|  |  |  | Incidence/prevalence of breast cancer [23] |
|  |  |  | Incidence/prevalence of diabetes [23] |
|  |  |  | Incidence/prevalence of Dementia/Alzheimer [23] |
|  |  |  | Incidence/prevalence of depression [23] |
|  |  |  | Incidence of Acute myocardial infarction [23] |
|  |  |  | Incidence of Stroke [23] |
|  |  |  | Prevalence of COPD (chronic obstructive pulmonary disease) [23] |
|  |  |  | Low birth weight [23] |
|  |  |  | Suicide attempt/death rate [23] |
|  |  |  | Perceived general health, age-standardised percentages of the population aged 16–84 with self-perceived health problems [23,24] |
|  |  |  | Injury rates by intent and sector, to include road traffic, workplace, home/leisure, suicide attempt, other violence [23] |
|  |  |  | Prevalence of any chronic illness or condition [23] |
|  |  |  | General musculoskeletal pain [23] |
|  |  |  | Psychological distress [23] |
|  |  |  | Difference in life expectancy at birth between EU Member States |
|  |  |  | and the EU average, by gender [25] |
|  |  |  | Euro Health Consumer Index—Outcomes sub-disciplines [35] |
|  |  | Health-related experience | - |
|  |  | Personal competences such as vision, hearing, verbal ability, memory and reasoning | Limitations in seeing, hearing, mobility, speaking, biting, agility [23] |
|  |  | Cognitive abilities | Incidence/prevalence of Dementia/Alzheimer [23] |
|  |  | Physical abilities | Limitations of usual activities, past 6 months, health-related [23] |
|  | Healthcare | Health coverage | Insurance coverage [23] |
|  |  |  | Physicians employed [23] |
|  |  |  | Nurses employed [23] |
|  |  |  | National Cancer Screening program [31] |
|  |  | Health system | Physicians employed, rate [23,24] |
|  |  |  | Nurses employed, rate [23,24] |
|  |  |  | Midwives employed, rate [24] |
|  |  |  | MRI units, CT (computed tomography) scans [23] |
|  |  |  | Waiting lists, for elective surgeries: PTCA (percutaneous transluminal coronary angioplasty), hip replacement, cataract operation [23] |
|  |  |  | Surgical wound infections [23] |
|  |  |  | Stage at cancer diagnosis [23] |
|  |  |  | Cancer survival rates; breast, cervix [23] |
|  |  |  | Nephropathy in diabetics: end-stage renal failure [23] |
|  |  |  | Number of general hospital beds per 100,000 inhabitants [24] |
|  |  |  | Euro Patient Empowerment Index [32] |
|  |  | Communication and assessment skills of people with whom individuals interact for health | - |
|  |  | Complexity and difficulty of the printed and spoken messages in the healthcare environment | - |
|  | Policy | Health promotion actions (education, social mobilization, advocacy) | Tobacco Control Scale [18,29] |
|  |  |  | Tobacco control policies, score considering seven policies [21] |
|  |  | Ability of the media, the marketplace, and governmental agencies to provide health information in an appropriate manner | - |
|  |  | Social support | Expenditure on social protection (% of GDP) [25] |
|  |  | Education system | Years of compulsory education [30] |
| Situational determinants (distal factors) |  | Social, environmental and political forces | Paved roads, % of the total road [19] |
|  |  |  | Pump price for gasoline [19] |
|  |  |  | Urban population, % of total population [19,20] |
|  |  |  | Internet access and broadband internet connections in households [36] |

B—Consequences.

| **Level** | **Sub-area** | **Area** | **Indicators** |
| --- | --- | --- | --- |
| Individual | Capacity to act independently on knowledge | Empowerment | Consumer Empowerment Index [33] |
|  | Motivation and self-confidence | Empowerment |  |
|  | Individual resilience | Empowerment |  |
|  | Ability to apply information to novel situations | Empowerment |  |
|  | Ability to participate in public and private dialogues about health, medicine, scientific knowledge and cultural beliefs | Participation  /Empowerment |  |
|  | Self-efficacy | Empowerment |  |
|  | Attitudes | Empowerment |  |
|  | Health knowledge (risk, diseases and treatments) | Empowerment | % Individuals (16–74) using the internet for seeking health information [34] |
|  | Health behaviors | Health behaviors | Smoking prevalence/rate [18,19,21,23]  The proportion of occasional smokers on the total number of current smokers [18,21,23]  Smoking rate Heaviness of Smoking Index [18,23]  Prevalence of e-cigarette users [21]  Percentage of 15-year-old males who smoke weekly and males aged 15 and over who smoke every day [25]  Obesity, % of population [19]  Percentage of 15-year-olds who were overweight/obese [25]  Body mass index [23]  Insufficient active, % of population [19]  Mean fasting blood glucose [19]  Mean systolic blood pressure [19,23]  Alcohol, total per capital consumption [19,20,23]  Western diet [19]  Teenage pregnancies [23]  Pregnant women smoking [23]  Alcohol: % of heavy drinkers, frequency of heavy drinking [23]  Use of illicit drugs [23] |
|  | Self-management skills/ability to care | Empowerment | - |
|  | Medical or medication treatment errors | Health behaviors | - |
|  | Compliance | Health behaviors | - |
|  | Health outcome | Health status/Health outcomes | UNAIDS (United Nations Programme on HIV/AIDS) global HIV indicators [17]  Homicide rate [20]  Healthy Life Years expectancy at birth stratified by sex [21,25]  Incidence rate of type 1 diabetes amongst children ≤ 14 years of age [22]  Death rates from infectious and parasitic diseases, age-standardized [22]  Death rates from respiratory infections, age-standardized [22]  Bacterial antibiotic susceptibility [22]  Life expectancy at various ages [23]  Life expectancy of men and women [25]  Infant mortality [23,25]  Perinatal mortality [23]  Standardised death rate, Eurostat 65 causes, age 0–65 [23]  Standardised death rate, Eurostat 65 causes, age 65+ [23]  Smoking-related deaths [23]  Alcohol-related deaths [23]  Drug-related deaths [23]  Incidence/prevalence of HIV/AIDS [23]  Incidence/prevalence of lung cancer [23]  Incidence/prevalence of breast cancer [23]  Incidence/prevalence of diabetes [23]  Incidence/prevalence of Dementia/Alzheimer [23]  Incidence/prevalence of depression [23]  Incidence of Acute myocardial infarction [23]  Incidence of Stroke [23]  Prevalence of COPD (chronic obstructive pulmonary disease) [23]  Low birth weight [23]  Suicide attempt [23]  Perceived general health, age-standardised percentages of the population aged 16–84 with self-perceived health problems [23,24]  Injury rates by intent and sector, to include road traffic, workplace, home/leisure, suicide attempt, other violence [23]  Prevalence of any chronic illness or condition [23]  General musculoskeletal pain [23]  Psychological distress [23]  Difference in life expectancy at birth between EU Member States and the EU average, by gender [25]  Euro Health Consumer Index- Outcomes sub-disciplines [32] |
|  | Hospitalization | Health services use | Average length of stay, limited diagnoses [23]  Hospital discharges, limited diagnoses [23]  Surgeries: PTCA (percutaneous transluminal coronary angioplasty), hip replacement, cataract operation [23] |
|  | Emergency care | Health services use |  |
|  | Healthcare costs | Health costs  /Sustainability | Health expenditure, % of the total expenditure [19,20]  Total public/private expenditures of health [23,35]  Private expenditures of health (% health expenditure) [23,35] |
|  | Use of healthcare services | Health services use | General practitioner utilization [23]  Outpatient visits [23]  Medicine use, selected items [23] |
|  | Patients/provider interactions | Participation |  |
|  | Healthcare access | Health services use/Equity | Self-reported unmet need for medical examination or Treatment [21] |
|  | Prevention/health promotion behaviors | Health behaviour | Condom use coverage [17]  Intake of fruit excluding juice [23]  Intake of vegetables [23]  Physical activity, time spent, energy expenditure [23]  Breastfeeding at various ages [23]  Vaccination coverage in children [23] |
|  | Screening behaviors | Health behaviour /Participation | Cancer screening coverage [23] |
|  | Improved capacity to influence social norms and interact with social groups | Empowerment/Equity | Individuals using the internet for interacting with public authorities [36] |
|  | Improved capacity to act on social and economic determinants of health | Empowerment/Equity | - |
| Community/  social | Social injustice | Equity/ Sustainability | Robbery rates [20]  Self-reported assault [20]  Assault rates [20]  Human Development Index (HDI) [37] |
